# Anti-Foc RT4 Activity of a Newly Isolated *Streptomyces* sp. 5–10 From a Medicinal Plant (*Curculigo capitulata*)

**DOI:** 10.3389/fmicb.2020.610698

**Published:** 2021-01-22

**Authors:** Tianyan Yun, Miaoyi Zhang, Dengbo Zhou, Tao Jing, Xiaoping Zang, Dengfeng Qi, Yufeng Chen, Kai Li, Yankun Zhao, Wen Tang, Jiaquan Huang, Wei Wang, Jianghui Xie

**Affiliations:** ^1^Key Laboratory of Biology and Genetic Resources of Tropical Crops, Ministry of Agriculture, Institute of Tropical Bioscience and Biotechnology, Chinese Academy of Tropical Agricultural Sciences (CATAS), Haikou, China; ^2^Haikou Experimental Station, Chinese Academy of Tropical Agricultural Sciences, Haikou, China; ^3^College of Tropical Crops, Hainan University, Haikou, China

**Keywords:** *Streptomyces*, banana wilt disease, antimicrobial activity, fermentation optimization, genome sequencing

## Abstract

Fusarium wilt of banana caused by *Fusarium oxysporum* f. sp. *cubense* (Foc) is a disastrous soil-borne fungal disease. Foc tropical race 4 (Foc TR4) can infect almost all banana cultivars. Until now, there is a shortage of safety and effective control methods and commercial banana cultivars with a resistance against Foc TR4. Biocontrol using environmentally friendly microbes is a promising strategy for the management of Foc TR4. Here, a strain 5–10, newly isolated from a medicinal plant (*Curculigo capitulata*), exhibited a high antifungal activity against Foc TR4. Combing the morphological characteristics and molecular identification, strain 5–10 was classified as a *Streptomyces* genus. The sequenced genome revealed that more than 39 gene clusters were involved in the biosynthesis of secondary metabolites. Some multidrug resistance gene clusters were also identified such as *mdtD*, *vatB*, and *vgaE*. To improve the anti-Foc TR4 activity of the strain 5–10 extracts, an optimization method of fermentation broth was established. Antifungal activity increased by 72.13% under the fermentation system containing 2.86 g/L of NaCl and 11.57% of inoculation amount. After being treated with the strain 5–10 extracts, the Foc TR4 hyphae shrinked, deformed, and ruptured. The membrane integrity and cell ultrastructure incurred irreversible damage. *Streptomyces* sp. 5–10 extracts play a fungicidal role in Foc TR4. Hence, *Streptomyces* sp. 5–10 will be a potential biocontrol agent to manage fungal diseases by exploring the microbial fertilizer.

## Introduction

Banana is the fourth major crop among the world’s developing countries (Shen et al., 2019). Fusarium wilt of banana caused by *Fusarium oxysporum* f. sp. *cubense* (Foc) seriously inhibited the development of banana industry (Ploetz, 2015). In particular, Foc tropical race 4 (Foc TR4) can infect almost all banana cultivars (Ghag et al., 2015). The disastrous disease had been found in major banana production areas of tropical and subtropical regions (Ghag et al., 2015; Ploetz, 2015). Until now, there are no commercial banana cultivars with an effective resistance against Foc TR4 (Shen et al., 2019). Compared with different prevention strategies, biocontrol using environmentally friendly microbes is considered as a promising strategy for management of the banana wilt disease (Bubici et al., 2019). Several reports have demonstrated the successful use of biocontrol agents against Foc TR4 (Ho et al., 2014).

Medicinal plants and their endophytes are important sources of bioactive compounds and secondary metabolites (Nimnoi et al., 2010). To date, only a few medicinal plants are investigated for their endophytic diversity and bioactive metabolites (Gouda et al., 2016). Especially, endophytic actinomycetes from medicinal plants can produce several novel compounds, including antibacterial, antifungal, antiviral, and antitumor drugs (Ahmad et al., 2017). These metabolites are widely applied in pharmaceutical and agricultural industries (Passari et al., 2015). Previous studies also showed that antifungal metabolites from *Streptomyces* sp. strain g10 and *S. noursei* Da07210 exhibited a strong antifungal activity against Foc TR4 (Getha et al., 2005; Wu et al., 2009).

In our present study, *Streptomyces* sp. 5–10 with a high antagonistic activity against Foc TR4 was newly isolated from the medicinal plant *Curculigo capitulata*. Its whole genome was sequenced to identify some key and novel gene clusters associated with the biosynthesis of secondary metabolites. To improve the production of bioactive metabolites of strain 5–10, the fermentation condition was optimized using a response surface method (RSM). After treatment with strain 5–10 extracts, morphology and ultrastructure of Foc TR4 mycelia were observed through scanning electron microscopy (SEM) and transmission electron microscopy (TEM), respectively. Hence, *Streptomyces* sp. 5–10 will be a potential bioresource for controlling banana wilt disease in future applications.

## Materials and Methods

### Sample Collection and Endophytic Actinomycetes Isolation

Roots, stems, and leaves of *C. capitulata* were collected from the “Wuzhishan” nature reserve (Latitude: 18°54′26′′ N, Longitude: 109°40′37′′ E) in Hainan, China. The selected samples were placed in sterile plastic bags and stored at 4°C.

To isolate the endophytic actinomycetes, the plant tissues were sterilized and thoroughly ground in a sterile mortar. Two hundred microliters of homogenate was added to a petri dish containing 50 mg/L of starch casein agar (SCA) actidione, nystatin antibiotic, nalidixic acid, and potassium dichromate (Lee et al., 2014). After incubation at 28°C for 30 days, colonies were isolated and identified according to the morphological characteristics, such as colonial morphology, color, and growth time. These isolates were kept at 4°C and sub-cultured on the yeast malt extract agar (ISP2) medium at intervals of 15 days.

### Phytopathogenic Fungi

*Fusarium oxysporum* f. sp. *cubense* tropical race 4 (Foc TR4, ATCC 76255) was conserved in our lab. A modified Foc TR4 strain overexpressing a green fluorescence protein (Foc-GFP) was provided by the Chinese Academy of Tropical Agricultural Sciences, Haikou, China. The pathogens were cultured on the potato dextrose agar (PDA) medium at 28°C for 3–5 days.

### Screening of Actinomycetes With Antifungal Activity

Antifungal activity of isolates against Foc TR4 were analyzed *in vitro* using the dual culture assay method (Yang et al., 2019). A circular piece of fungal agar, already grown (5 mm diameter), was placed in the center of a plate. A circular piece of actinomycetes agar, already grown (5 mm diameter), was inoculated on one side at about 2.5 cm from the plate center. A fungal piece of Foc TR4 alone was used as a control. Antifungal activity was recorded after 7 days of cocultivation at 28°C. The growth diameters of Foc TR4 were measured by a cross method (Sadeghian et al., 2016). The inhibition rate of mycelial growth was calculated using the following formula: (diameter of untreated colony - diameter of treated colony)/diameter of untreated colony × 100%.

### Morphological and Biochemical Characteristics of Strain 5–10

The morphological, biochemical, and physiological characteristics of strain 5–10 were determined by the classification status of the selected actinomycetes strain (Ahmad et al., 2017). The growth profiles were recorded on six different media (Shirling and Gottlieb, 1966). The morphology of the isolate was detected by a scanning electron microscopy (SEM, model S-4800, Hitachi Limited, Japan). After 8 days of growth on the ISP2 medium, mycelial structure and spore surface were observed by SEM. Some indices including cellulose, starch, and gelatine of hydrolysis, production of H_2_S, nitrate reduction, and urease activity were measured according to the description of Shirling and Gottlieb (1966). Effects of pH (4–10), carbon utilization, nitrogen utilization, and NaCl tolerance on the strain growth were also assayed (Ahmad et al., 2017).

### Genome Sequencing and Functional Annotation

The whole genome of the selected actinomycete was sequenced using Illumina Hiseq × 10 platform by the Majorbio Bio-pharm Technology Co., Ltd, China. Genomic DNA library was constructed by the TruSeqTM DNA Sample Prep Kit (Illumina). Reads were assembled using the SOAP *de novo* software v2.04. Reads with >10% Ns and/or 25–35 bases of low quality (<Q20) were filtered out. Adapter sequence and duplication contamination were also removed (Cao et al., 2017). Encoding genes, tRNAs, and rRNAs were predicted by the Glimmer v3.02^1^, tRNAscan-SE v2.0^2^, and Barrnap v0.8^3^ softwares, respectively. The sequence similarity of actinomycete genome was aligned using blastp (BLAST+, v2.3.0). A circular map of pairwise genome was generated and visualized by the Circos v0.69-6 and CGview v2. The 16S rRNA sequence obtained from the strain genome was used for performing a phylogenetic analysis. The sequence was aligned against a public database using the EzBioCloud tool^4^. A phylogenetic tree was constructed using the neighbor-joining method of MEGA 7.0. Gene prediction and annotation were carried out by the prokaryotic genome annotation pipeline of NCBI (Paungfoolonhienne et al., 2014). The anti-SMASH program was used to search putative gene clusters responsible for biosynthesis of secondary metabolites in the selected actinomycete genome (Olano et al., 2014).

### Fermentation Condition Optimization of Actinomycete

#### Fermentation Optimization Design

In order to obtain a high antifungal activity of actinomycete extracts, a response surface methodology (RSM) was used to optimize the reaction condition, including the Plackett–Burman design, the path of steepest ascent design, and the Box–Behnken design (Kumari et al., 2016). Actinomycete was inoculated with 100 ml of sterilized liquid medium of soybean at 28°C. The fermentation broth was extracted with ethanol (filtrate: ethanol = 1:1, v/v). The solvent was removed using a rotary vacuum evaporator R 206D (SENCO, Shanghai, China). A brown residue was obtained and stored at 4°C. Antifungal activity was determined by an agar well diffusion method.

#### Plackett–Burman Design

A set of 12 experimental runs with different combinations of independent variables was generated using a Design-Expert software (Version 10.0, Stat-Ease Inc., Minneapolis, United States). Nine parameters with two different levels (−1, 1) included soluble starch (g/L) (*X*_1_) (20, 25), soy flour (g/L) (*X*_2_) (15, 18.75), yeast extract (g/L) (*X*_3_) (5, 6.25), peptone (g/L) (*X*_4_) (2, 2.5), NaCl (g/L) (*X*_5_) (4, 5), initial pH (X_6_) (8, 10), fermentation time (d) (*X*_7_) (8, 10), shaker speed (rpm) (*X*_8_) (200, 250), and inoculation amount (%) (*X*_9_) (6, 7.5). All experiments were performed in triplicate. The Plackett–Burman experiment was designed in the light of the first-order polynomial model (Surwase et al., 2012):

Y=β⁢0⁢+β⁢i⁢x⁢i

where *Y* was the response, β_0_ was the model intercept, β_*i*_ was the regression coefficient, and *X*_*i*_ was the level of independent variable.

#### Path of Steepest Ascent Design and Box–Behnken Design

Based on the above results obtained from the Plackett–Burman experiment, the step length and direction were calculated in the path of the steepest ascent design (Yang et al., 2017). The Box–Behnken design (BBD) was performed to enhance the active production of metabolites and determine the optimal value of significant variable (Surwase et al., 2012). NaCl (2.7, 2.9, 3.1 g/L) and inoculation amount (11.0, 11.9, and 12.8%) were set at three different levels (−1, 0, +1). Nine experiments were carried out to optimize these key factors. A multiple regression was calculated for obtaining a model of the most significant factor. Each response was fitted with an independent second-order polynomial model (Kumari et al., 2016):

Y=β⁢0+β⁢i⁢x⁢i+β⁢ii⁢x⁢i⁢x⁢j+β⁢i⁢j⁢x⁢i⁢x⁢j

where *Y* was the antifungal activity (predicted response), *x*_*i*_ and *X*_*i*_ represented the independent variables, β_0_ was an intercept, β_*i*_ was a linear coefficient, and β_*ii*_ was a quadratic term coefficient. The relationship between coded value and actual value was calculated as follows:

$$x_{\text{i}}=\frac{X_{\text{i}}-X_{\text{o}}}{\delta \,X}$$

where *X*_0_ was the natural variable at the center point, and *δX* was the value of step change.

The fitness of data in the equation was validated with a coefficient variation (*R*^2^) in the statistical analysis of variance (ANOVA). The authenticity of the model was evaluated by the predicted value for active metabolites under the optimized condition (Arora et al., 2015). Data were statistically analyzed using the Design-Expert version 8.0 (Stat-Ease Inc., Minneapolis, MN, United States) (Arora et al., 2015). Results of BBD were verified by performing an experiment according to the predicted conditions (Kumari et al., 2016). All experiments were performed in triplicate.

### Effect of Actinomycete Extracts on Spore Germination of Foc RT4

Inhibitory efficiency of actinomycete extracts on spore germination of Foc TR4 was determined according to the description of Huang et al. (2011). Equal volume of fungal spore suspension (10^5^ cfu/ml) and actinomycete extracts was mixed and co-incubated at 28°C. The extracts were replaced with sterile water and was used as a control. After incubation for 6 h, 10 μl of the mixture was dropped on a sterile glass slide. Among the detected 200 conidia, the number of germinated spores was counted by a light microscope (model Axio Scope A1, Carl Zeiss AG, Germany). The inhibition percentage of spore germination (I) was calculated using the following formula: *I*(%) = [(Nc − Nt)/Nc] × 100, where Nc and Nt represented the number of germinated spores in the control and treatment groups, respectively (Alijani et al., 2019). Three replicates were performed for each treatment.

### Antagonistic Effects of Actinomycete Extracts on Mycelial Growth of Foc-GFP *in vitro*

Inhibition ability of extracts to mycelial growth of Foc-GFP was detected using a modified “cross-plug” method (Getha et al., 2005). Briefly, strain 5–10 extracts were added to a sterilized cover slip (1 cm × 1 cm) with the growing Foc-GFP mycelia. After incubation at 28°C for 2–3 days in the dark, the mycelial samples were observed using a light microscope (model Axio Scope A1, Carl Zeiss AG, Germany) every 2 days.

### Effect of Actinomycete Extracts on Mycelial Morphology of Foc TR4

Actinomycete extracts were added to the PDA medium with a final concentration of 500 μg/ml. Foc TR4 was inoculated and cultured on the plate at 28°C for 5 days. The plate without extracts was used as a negative control. Foc TR4 mycelia were collected and fixed overnight at 4°C with 2.5% (v/v) of glutaraldehyde. After rinsing twice using 0.1 mol/L of phosphate buffer saline (PBS, pH 7.4), the mycelial sections were dehydrated with a gradient of ethanol solution (30, 50, 70, 80, 90, 95, and 100%) for 20 min, and then, ethanol was replaced with isoamyl acetate. The dried samples were coated with a gold-covered method (Xing et al., 2014). Mycelial morphology of Foc TR4 was observed by SEM (model S-4800, Hitachi Limited, Japan).

### Effect of Actinomycete Extracts on Ultrastructure of Foc TR4 Cells

Foc TR4 was inoculated on the PDA medium with 500 μg/ml of strain 5–10 extracts at 28°C for 5 days. The Foc TR4 mycelia were fixed with glutaraldehyde (2.5%, v/v) overnight at 4°C and postfixed using osmium tetroxide (1%, v/v). After washing three times with PBS (0.1 mol/L, pH 7.0), the samples were dehydrated with different gradients of ethanol solution and embedded in the Epon 812 resin at 37°C for 12 h, 45°C for 12 h, and 60°C for 24 h, respectively (Xing et al., 2014). The Foc TR4 mycelia were sliced by an ultra microtome (Leica, UC6 CM1950, Germany) and stained with uranyl acetate and citric acid for 30 min, respectively. The ultrastructure of transverse Foc TR4 mycelia was detected by TEM (JEM-1400 Flash, Hitachi Limited, Japan).

### Effect of Actinomycete Extracts on Cellular Electrolyte Leakage of Foc RT4

Electrolyte leakage of Foc RT4 cells was used to evaluate the effects of actinomycete extracts on cellular leakage of Foc RT4 according to the description of Xu et al. (2019). It was calculated by the formula [(J_1_ − J_0_/J_2_ − J_0_)] × 100%. J_1_ represented the value of extracellular conductivity at 1, 2, 4, 6, 12, or 24 h after extract treatment. The conductivity values of boiled and untreated samples were taken as J_2_ and J_0_, respectively. The electrical conductivity was measured using a conductivity meter (DDS-307, Hanghai Yueping Scientific Instrument Co. Ltd., Shanghai, China).

### Effect of Actinomycete Extracts on Plasma Membrane Integrity of Foc RT4

Propidium iodide (PI) of cell membrane impermeable fluorescent dye was applied to investigate plasma membrane integrity of Foc RT4 cells treated with actinomycete extracts (Zhang et al., 2020). Spore suspension of Foc RT4 (1 × 10^5^ cfu/ml) was mixed with different concentration (250 μg/ml, 500 μg/ml) extracts and co-incubated at 28°C for 4 h (Xu et al., 2019). The untreated sample was used as a control. The treated cells were washed twice with PBS, stained with PI for 5 min at 28°C in the dark, and finally detected by a laser confocal scanning microscope (Olympus corporation, FV1000, Tokyo, Japan).

## Results

### Isolation and Identification of Actinomycetes With a Strong Antifungal Activity Against Foc TR4

**FIGURE 1:**
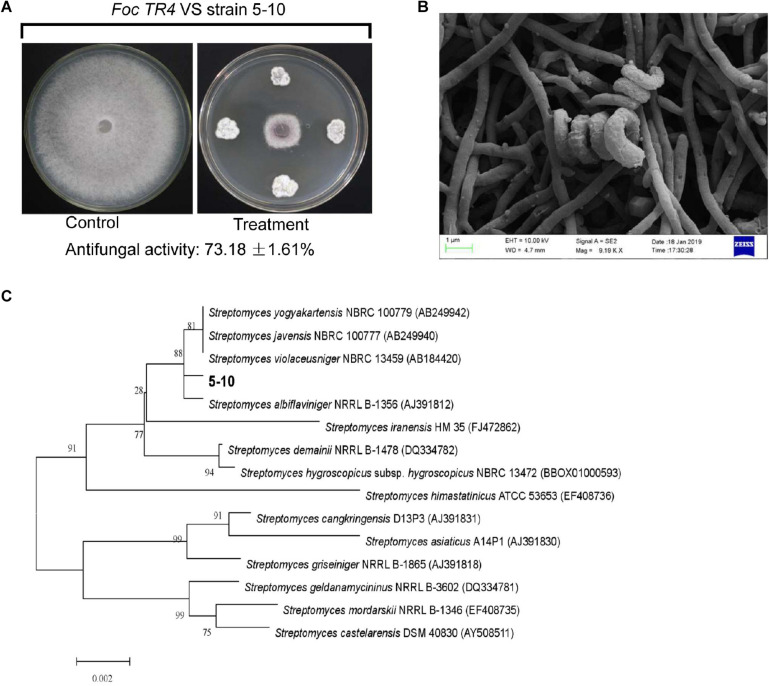
Isolation and identification of strain 5–10. **(A)** Isolation of strain 5–10 with a high antifungal activity against *Fusarium oxysporum* f. sp. *cubense* tropical race 4 (Foc TR4). **(B)** Morphological characteristics of strain 5–10 spores using scanning electron microscopy (SEM). **(C)** Construction of phylogenetic tree using the 16S rRNA sequences from different *Streptomyces*.

A total of 16 different endophytic actinomycetes based on their antifungal activities against Foc TR4 were isolated from different tissues of *C. capitulate*. Out of them, 25% of these isolates displayed above 49.25% of antifungal activity. Especially, an isolate labeled with 5–10 had 73.18% of growth inhibition rate against Foc TR4 (Figure 1A). By contrast, strain 5–10 can grow well on the ISP2 or ISP3 medium, and no pigment was observed on all the tested media (Supplementary Table 1). The spiral spore chain and fold spore surface was detected by SEM (Figure 1B). The physiological and biochemical characteristics of strain 5–10 were evaluated by analyzing different enzyme products, nitrogen and carbon utilization, and growth ability on different pH media (Supplementary Table 2). It cannot only utilize cellobiose, soluble starch, sorbitol, and melibiose as carbon source, but also use valine, serine, histidine, and phenylalanine as nitrogen source. Based on morphological, biochemical, and physiological analyses, strain 5–10 has a typical profile of *Streptomyces* genus (Williams et al., 1983).

### Genome Sequencing and Annotation of Strain 5–10

#### Genome Sequence Data of Strain 5–10

**FIGURE 2:**
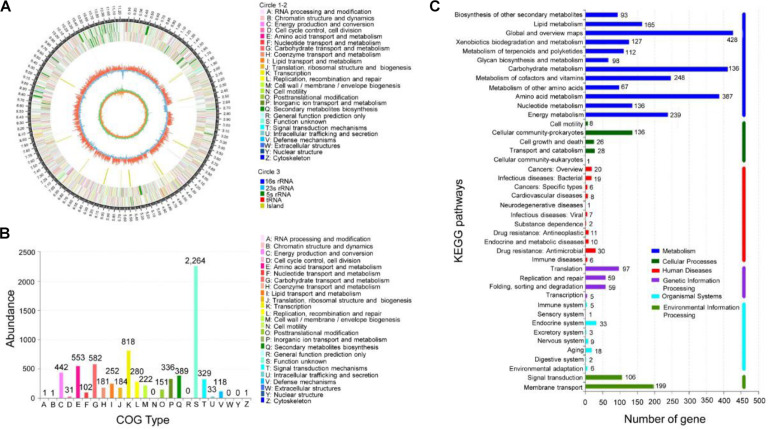
Genomic information of strain 5–10 and functional annotation of predicted genes. **(A)** Genomic map of strain 5–10 by sequencing. **(B)** COG functional classification. **(C)** KEGG classification of metabolic pathways.

To assess the production potential of secondary metabolites of *Streptomyces* sp. 5–10, the genome mining method was efficient for identifying biosynthetic gene clusters and predicting bioactive compounds (Arn et al., 2020). The sequenced genome of strain 5–10 produced a total base pair of 1,612,997,704 bp. After removing the Illumina PCR adapter reads and low-quality reads, a total of 4,759,815 mate pair reads (total 1,433,113,234 bp) were obtained. The size of the complete genome was 9,528,477 bp, and the G + C content was 71.63%. There were 273,308 bp of the repeat sequences predicted by the Tandem Repeats Finder tool. The sequences of strain 5–10 were deposited in the GenBank database with an accession number JACVYG000000000. A total of 9,971 genes were predicted with a total length of 9,528,477 bp, constituting 84.62% of the entire assembled genome (Figure 2A). Seventy-five *tRNA* genes and four *rRNA* genes were identified. A 16S rDNA sequence of 1,493 bp was deposited in the GenBank database of NCBI with an accession number MK356358. After the phylogenetic analysis, the 16S rDNA sequence exhibited a high similarity with *S. albiflaviniger* NRRL B-1356 (Figure 1C). However, the genome sequence of *S. albiflaviniger* was not found in the GenBank database, so the average nucleotide identity (ANI) value cannot be calculated to evaluate the genetic relatedness.

#### Functional Annotation of Strain 5–10 Genome

The encoding gene sequences were aligned with the COG and KEGG databases to predict the putative gene functions and the metabolic pathways. Among them, 7,155 genes were successfully annotated with COG (Figure 2B), accounting for 71.76% of all genes. Annotation analysis showed that these genes were mainly involved in secondary metabolite biosynthesis, transport and catabolism (389 genes), amino acid transport and metabolism (553 genes), carbohydrate transport and metabolism (582 genes), energy production and conversion (442 genes), coenzyme transport and metabolism (181 genes), lipid transport and metabolism (252 genes), translation, ribosomal structure, and biogenesis (184 genes), transcription (818 genes), defense mechanisms (118 Genes), etc. The KEGG annotation indicated that 3,174 genes were involved in the metabolism of terpenoids and polyketides (112 Genes), biosynthesis of other secondary metabolites (93 genes), xenobiotic biodegradation and metabolism (127 genes), glycan biosynthesis and metabolism (67 genes), carbohydrate metabolism (412 genes), and amino acid metabolism (387 genes) (Figure 2C). Notably, a large number of unknown functional genes identified may be involved in the biosynthesis regulation of secondary metabolites.

#### Gene Clusters of Secondary Metabolite Biosynthesis

**FIGURE 3:**
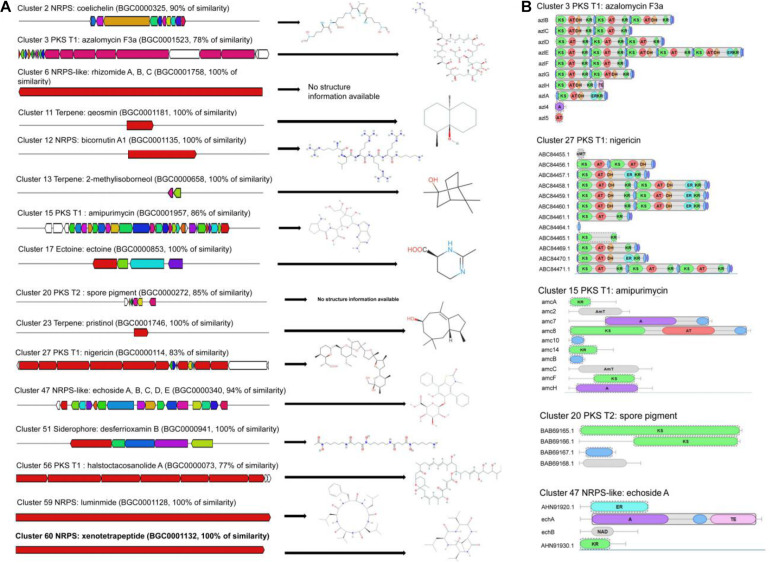
Biosynthetic gene clusters and core structures predicted by AntiSMASH. **(A)** The putative biosynthetic gene clusters responsible for synthesis of antimicrobial compounds. **(B)** The core structure characteristics of azalomycin F3a, amipurimycin, nigericin, spore pigment, and echoside A in the strain 5–10 genome.

A total of 60 gene clusters responsible for secondary metabolites were predicted using the Anti-SMASH program (Supplementary Table 3). Especially, 19 gene clusters exhibited more than 70% of similarity with the known sequences, including five non-ribosomal peptide synthase (NRPS) gene clusters, four NRPS-like gene clusters, six polyketide synthase gene clusters (five PKS-T1 and one PKS-T2), one siderophore gene cluster, two terpene gene clusters, and one ectoine gene cluster (Figure 3A). Especially, other gene clusters showed 100% of similarities with NRPS, including luminmide of *Photorhabdus laumondii* TTO1, xenotetrapeptide of *Xenorhabdus nematophila* ATCC 19061, bicornutin A1 of *Xenorhabdus budapestensis*, NRPS-like (rhizomide A of *Paraburkholderia rhizoxinica* HKI 454 and 2-methylisoborneol of *S. griseus* NBRC 13350). Compared with other genomes of *Streptomyces*, 100% of similarity was also observed in two terpene gene clusters (geosmin of *Streptomyces coelicolor* A3 and pristinol of *Streptomyces pristinaespiralis* ATCC 25486), one ectoine gene cluster (ectoine of *Streptomyces anulatus*), and one siderophore gene cluster (desferrioxamin B of *Streptomyces griseus* NBRC 13350). Two gene clusters had 83% of similarity with one PKS-T1 gene cluster (nigericin of *Streptomyces violaceusniger*) and one PKS-T2 gene cluster (spore pigment of *Streptomyces avermitilis*).

#### Core Structures of Biosynthetic Gene Clusters

By contrast, the predicted core structures of five gene clusters exhibited more than 70% of similarity with the known gene clusters (Figure 3B). The strain 5–10 genome contained three potential PKS-T1 clusters (cluster 3, cluster 5, and cluster 27). Cluster 3 had 78% of similarity with PKS-T1 of *Streptomyces* sp. 211726 known as the production of antimicrobial azalomycin F3a (Xu et al., 2017). Cluster 15 showed 86% of similarity with PKS-T1 of *Streptomyces novoguineensis* responsible for producing antimicrobial amipurimycin (Kang et al., 2019). Cluster 27 had 83% of similarity with PKS-T1 from *S. violaceusniger*, which participated in the biosynthesis of antibacterial compounds such as nigericin (Harvey et al., 2007). The PKS module consisted of ketosynthase (KS), acyltransferase (AT), ketoreductase (KR), enoylreductase (ER), or dehydratase (DH) domain. Three PKS clusters demonstrated a distinct difference of core structures. Similar results were also observed in the number and type of other biosynthetic, transport, and regulatory genes (Figure 3B). These results suggested that the novel and diverse compounds were potentially produced by strain 5–10. Further experiments need to be performed to identify the biosynthetic gene clusters producing the antifungal compounds of strain 5–10.

### Optimization of Fermentation Conditions Using RSM

#### Determination of Key Factors in the Growth Medium of Stain 5–10

**FIGURE 4:**
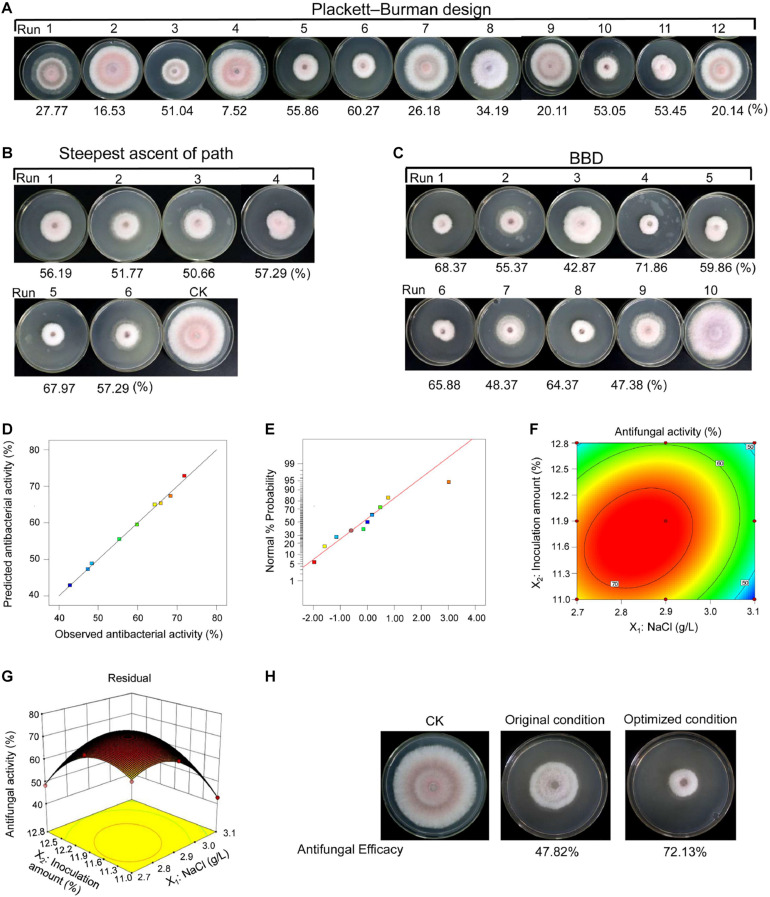
Fermentation condition optimization of strain 5–10 by RSM. **(A)** The Placket–Burman design; **(B)** the steepest ascent of path; **(C)** the Box–Behnken design (BBD). **(D)** Diagnostic plots of the observed values and predicted values showing the model reliability. **(E)** Normal probability of internally studentized residuals. **(F)** Response surface plots of antifungal activity. **(G)** Contour plots of antifungal activity. **(H)** Comparison of antifungal activity before and after optimization.

The medium composition and culture conditions were optimized by RSM to improve the production of bioactive metabolites and discover some important active compounds. The Plackett–Burman design was applied to evaluate the effects of some growth factors (such as soluble starch, soy flour, yeast extract, peptone, NaCl, initial pH, shaker speed, fermentation time, and inoculation amount) on antifungal activity of strain 5–10 (Figure 4A and Supplementary Table 4). The limited variables and the variance (ANOVA) analysis are demonstrated in Supplementary Table 5. Based on these data, a regression equation was obtained as follows:

$$\begin{array}{c} Y=35.50917+5.36417\,\mathrm{X1}+2.99917\,\mathrm{X2}-3.1575\,\mathrm{X3}+5.83917\,\mathrm{X4}\\ -8.17583\,\mathrm{X5}+0.8575\,\mathrm{X6}+4.6625\,\mathrm{X7}+3.68917\,\mathrm{X8}+10.65083\,\mathrm{X9} \end{array}$$

A coefficient determination (*R*^2^ = 97.67%) suggested that the regression equation was suitable for predicting antifungal activity of strain 5–10 extracts during fermentation. The *t*-test and *p*-value were used to evaluate the effects of different growth factors on metabolite production. By contrast, the NaCl concentration and inoculation amount were considered as two key factors (*p <* 0.05). The inoculation amount had a positive relationship with antifungal activity of fermentation products, while the NaCl concentration exhibited a negative relationship. Therefore, NaCl and inoculation amount were selected for further optimization to obtain a maximal production of active metabolites.

#### Analysis of Significant Variables Using the Path of Steepest Ascent Design and Box–Behnken Design

According to the results of the Placket–Burman design, these key factors were further identified by the path of the steepest ascent. The greatest antifungal activity was observed in the fifth group (Figure 4B and Supplementary Table 6). The BBD was used to determine the optimal levels of NaCl and inoculation amount. According to antifungal activities of strain 5–10 extracts under different fermentation conditions, an equation was predicted as follows:

$$Y=71.70-5.92\,X\,1-2.92\,X\,2-5.13\,X\,1\,X\,2-10.74\,X\,1^{2}-9.746\,X\,2^{2}$$

where *Y* was the antifungal activity, and *X*_1_ and *X*_2_ represented the NaCl concentration and the inoculation amount, respectively.

The actual and predicted values of antifungal activities are shown in Supplementary Table 7 and Figure 4C. The predicted values were consistent with the observed values in the operating range of variables (Figure 4D). The residual plots and linear patterns represented normality in the error term (Figure 4E) (Wang et al., 2008). The elliptical contours showed that a significant interaction existed between NaCl and inoculation amount in the three-dimensional response plot (Figures 4F,G). A statistical significance was analyzed to evaluate the feasibility of the model equation (Supplementary Table 8). Based on *p*-value (0.0007), *F*-value (164.68), coefficient of determination (*R*^2^ = 0.99903), and coefficient of variation (1.74%), the model was suitable for predicting antifungal activity of strain 5–10 extracts (Surwase et al., 2012). In comparison with the interaction of NaCl and inoculation amount, the binomial coefficients (*X*_1_^2^ and *X*_2_^2^) of regression equation were obviously different, suggesting that the effect of these two factors on antifungal activity was not only a simple linear relationship.

According to the predicted model, the maximal antifungal activity (74.03%) was obtained under the fermentation condition with 2.838 g/L of NaCl and 11.706% of inoculation amount, and then, an experiment was performed to test the reliability of the predicted model. Under the optimized condition, strain 5–10 extracts had a higher antifungal activity (72.13%) than that before optimization (47.82%) (Figure 4H). Although the observed value was a minor difference with the predicted result, less than 10% of difference can be considered as the validity of the model (Levin et al., 2008). Hence, the constructed model was reliable and reproducible in the present study.

### Effect of Strain 5–10 Extracts on Spore Germination, Mycelial Morphology, and Ultrastructure of Foc TR4 *in vitro*

**FIGURE 5:**
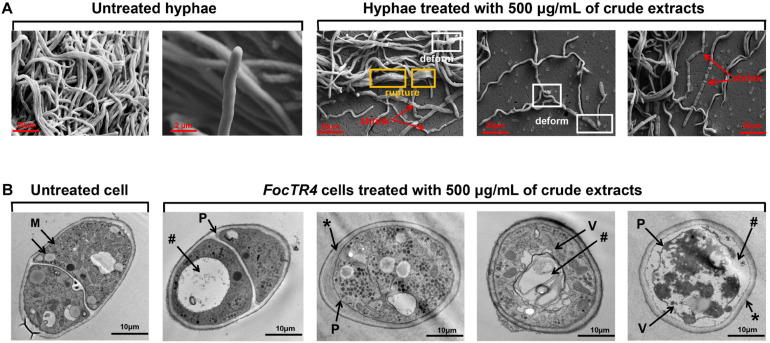
Effect of strain 5–10 extracts on structural characteristics of Foc TR4. Strain 5–10 extracts were mixed with the potato dextrose agar (PDA) medium and inoculated at 28°C for 5 days. **(A)** Morphological characteristics of Foc TR4 hyphae treated with 500 μg/ml of extracts by SEM. The red arrows showed the wizened hyphae of Foc TR4, the yellow boxes represented the ruptured hyphae of Foc TR4 and the white boxes exhibited the deformed hyphae. **(B)** Ultrastructure of the transverse Foc TR4 hyphae treated with 500 μg/ml of extracts by transmission electron microscopy (TEM). M, mitochondria; *, thickened and irregular cell walls; P, plasmolysis; V, matrix loss in vesicles; #, vacuolization.

Strain 5–10 extracts significantly decreased spore germination of Foc RT4 compared to 80% of germinated spores detected after 6 h in the control group. Only 27.35% of the germination rate was observed after treatment with the strain 5–10 extracts (Supplementary Figure 1). Moreover, the cell wall of untreated Foc TR4 had a linear and smooth structure (Figure 5A). Intact mitochondria (M), vesicles (V), and lipid bodies in Foc TR4 cells were clearly observed in the control group (Figure 5B). After treatment with 500 μg/ml of extracts, the hyphae became wizened and ruptured. A large number of vacuoles and disintegrated cytoplasm were found in the cell matrix. Obvious vacuolization was also detected. Additionally, Foc TR4 overexpressing a *GFP* gene was used to further analyze the effect of extracts on the mycelial morphology by the fluorescence microscope. Most of GFP-Foc4 hyphae became dissolved, and fluorescence signals disappeared (Supplementary Figure 2). Therefore, this interference was sporistatic by inhibiting the formation of germ tubes and hyphal growth.

### Effect of Extracts on Membrane Permeability of Foc RT4 Cells

**FIGURE 6:**
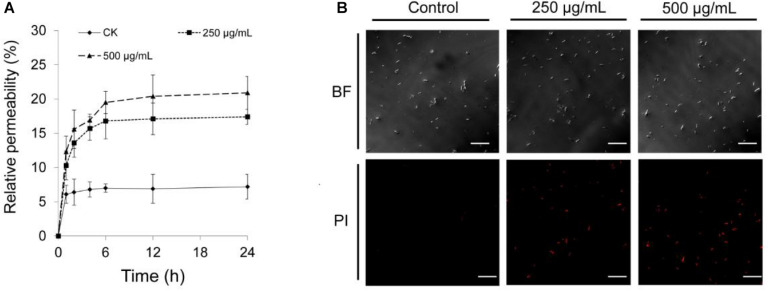
Effect of strain 5–10 extracts on cell membrane integrity of Foc TR4. **(A)** Measurement of Foc TR4 extracellular conductivity after treated with strain 5–10 extracts. **(B)** Red fluorescence signal showing the disrupted plasma membranes of Foc TR4 spores stained with PI. Scale bars = 200 μm.

The electric conductivity of Foc TR4 cells was increased by 24 and 29% after treatment with 250 and 500 μg/ml^–1^ of extracts for 12 h, respectively (Figure 6A). The PI staining was used to further detect the plasma membrane integrity of cells. As shown in Figure 6B, Foc TR4 spores cannot be stained by PI, or low red fluorescence was observed in the control group. In the treatment group, obvious fluorescence signals of PI were observed. Along with the increase in the extract concentrations, the spores with red fluorescence signal were gradually increased, suggesting that Foc TR4 plasma membrane was seriously broken by strain 5–10 extracts.

## Discussion

Fusarium wilt of banana was prevalent in most of major banana production areas in tropical and subtropical regions (Ghag et al., 2015; Ploetz, 2015). Some beneficial microbes such as *Trichoderma* spp., *Pseudomonas* spp., and *Bacillus* spp. had been used to biocontrol banana wilt disease (Bubici et al., 2019). However, a large variation of biocontrol efficiency was frequently observed due to the selected microbes (Zhu et al., 2020). Until now, very few studies are reported for the biocontrol of banana fusarium wilt using *Streptomyces* species (Bubici et al., 2019; Zhu et al., 2020). In our recent study, an endophytic actinomycete 5–10 with a high antifungal activity against Foc RT4 was isolated from roots of *C. capitulata*. Previous studies showed that actinomycetes from different medicinal plants produced more bioactive metabolites with a potential application value in the agricultural and medical fields (Gouda et al., 2016). It might be because that actinomycete metabolites may be related with medicinal properties of host plants. For example, some compounds isolated from *C. capitulata* showed activities of antitumor and immunosuppression (Nie et al., 2013). Some isolates from *Streptomyces* genus were also prolific producers of antimicrobial compounds (Seipke et al., 2012). These results suggested that strain 5–10 will be a promising biocontrol agent against banana wilt disease.

Antifungal activity of the strain 5–10 extracts was supported by the gene clusters responsible for secondary metabolites within the strain 5–10 genome. In the strain 5–10 genome, the identified 60 biosynthetic gene clusters proved a high potential for producing diverse chemical compounds. Notably, 47 gene clusters encoding PKS and NRPS were identified based on functional active domains, which were important members for contributing to the biosynthesis of major secondary metabolites (Moolhuijzen et al., 2020). The prevalence of NRPS and PKS clusters of strain 5–10 is distinguished from other *Streptomyces* strains. Previous studies indicated that PKS and NRPS were large multifunctional enzymes with various catalytic domains, which were responsible for the production of some active metabolites in actinomycetes (Minowa et al., 2007). PKS can utilize a variety of bioavailable acyl to build blocks (for example, acetate, propionate, butyrate, etc.) and iterative decarboxylative claisen condensation to generate stereoenriched enzyme-bound polyketide chains, which can be further enzymatically tailored to yield the final bioactive products (Riva et al., 2014). We also found that the NRPS-like echoside A belonging to the ANL superfamily of adenylating enzymes participated in catalyzing the partial adenylation half reaction (Singh et al., 2017). Interestingly, some identified gene clusters of *NRPS* and *PKS* lacked homologs against the anti-SMASH database. For example, telomycin (BGC0001406) and daptomycin (BGC0000336) demonstrated less than 5 and 10% of similarity in comparison with the known function genes. It suggested that these biosynthetic gene clusters might lead to the production of novel compounds (Siupka et al., 2020).

Additionally, other bioactive metabolites were also identified in the strain 5–10 genome, including antibiotics, toxins, siderophores, and immunosuppressive agents such as coelichelin, rhizomide A, meridamycin, amipurimycin, nystatin, azalomycin F3a, etc. Miharamycin was a peptidyl nucleoside antibiotic, showing a remarkable activity against the rice blast disease (Kang et al., 2019). As a ferric-iron-chelating peptide, coelichelin effectively harnessed the microbe’s own cellular machinery to improve cellular uptake and delivery of the antimicrobial compounds (Williams et al., 2019). Nigericin showed strong antibacterial and anticancer activities by moving sodium and potassium ions, resulting in changes in the ion gradient in the energetic metabolism (Ortega et al., 2019). Moreover, genes responsible for indole biosynthesis and ion acquisition in the strain 5–10 genome might also play important roles in the biocontrol of Foc TR4. A previous study showed that the indole skeleton displayed a broad spectrum of antimicrobial bioactivity (Qin et al., 2015). Siderophores produced by *Streptomyces* were involved in the growth inhibition of phytopathogen by depriving some essential ions (Zhu et al., 2019). Actually, the biosynthetic pathways of strain 5–10, which most likely originated from *Proteobacteria*, were more complicated due to some unknown biosynthetic genes and regulatory genes (Paulus et al., 2017). The high number of biosynthetic gene clusters influences the metabolic potential of microbes for improving the competition for nutrients in the environment (Chevrette et al., 2019). Hence, *Streptomyces* sp. 5–10 was an excellent candidate for the biocontrol of phytopathogenic fungi based on its genome profile.

Biosynthesis of multifarious compounds in bacteria was closely related to the fermentation condition (Bretschneider et al., 2013). Due to a complex and non-linear growth process of a microbe, a minor variation in the fermentation media can significantly influence the active compound yield and metabolic profile (Kaur et al., 2014). In our study, the medium composition and culture conditions were optimized by RSM. By contrast, NaCl and inoculation amount were two key factors. Accumulated evidence indicated that an appropriate concentration of NaCl can increase production of microbial antibiotics by mediating the osmotic pressure of the medium (Pelczar et al., 1993). When osmolarity in the medium was reduced by the decrease in NaCl, strain 5–10 might drastically promote the production of secondary metabolites. It was supported by the fact that strain 5–10 cultured on the medium with 2.84 g/L of NaCl had the highest antifungal activity (72.13%). In addition, inoculation amount was approved to be related closely with metabolite production in our study. Insufficient inoculation amount may lead to low biomass of active metabolites, while a higher inoculation amount caused accumulation of toxic substances (Wang et al., 2008; Noura et al., 2013). Therefore, the antifungal activity increase of strain 5–10 fermentation broth might be related to the improvement of active compounds or the change in metabolic profiles in the optimized medium. Chiani et al. (2010) improved the production of a bioactive desferrioxamine B of microbe by slight addition of NaCl. Jiang and Huang (2004) reported that the increase in inoculation amount resulted in the significant decrease in antimicrobial and antifungal azamycin. So it is significant to improve the antifungal activity of strain 5–10 extracts with a short-cycle and low-cost fermentation method.

Compared with *Streptomyces* sp. strain g10 (Getha et al., 2005) and *S. noursei* Da07210 (Wu et al., 2009), *Streptomyces* sp. 5–10 extracts decreased conidial germination and destroyed mycelium structures of Foc TR4. It was due to the rupture of cell membrane and the degradation of cell walls. A previous study showed that chitinase synthesized by *Streptomyces lydicus* WYEC108 could hydrolyze components of fungal cell wall *in vivo* (Mahadevan and Crawford, 1997). In addition, the degenerated cell organelles and large vacuoles were observed in Foc TR4 cells treated with the extracts. The red fluorescence signal of PI was visibly enhanced with the increase in extract amount, suggesting that the phenomenon of cell death occurred in a large number of Foc TR4 cells (Xu et al., 2019). It was further supported by the result that extracellular conductivity of fungal cells increased rapidly after treated with strain 5–10 extracts. Similarly, *Streptomyces* ma. FS-4 caused the plasma membrane destruction and cell apoptosis of Foc TR4 (Duan et al., 2020). Actually, the integrity of cytoplasmic membrane is one of crucial factors for various essential functions of microbes (Huang et al., 2003). Thus, strain 5–10 may exert its antifungal activity against Foc TR4 by dissolving the cell wall and damaging cytoplasmic membrane and cell ultrastructure.

## Conclusion

A strain *Streptomyces* sp. 5–10 with a high antifungal activity of Foc TR4 was newly isolated from roots of *C. capitulata*. A total of 60 putative gene clusters responsible for antimicrobial metabolite biosynthesis were predicted in the sequenced genome of strain 5–10. Some antimicrobial genes were also identified by alignment with databases. Furthermore, the RSM was applied to optimize the fermentation condition to enhance antifungal activity of strain 5–10 extracts. By contrast, the NaCl concentration and inoculation amount were considered as two-key fermentation parameters. The strain 5–10 extracts caused a shrunken and ruptured morphology of Foc TR4 hyphae and the membrane permeability. Hence, *Streptomyces* sp. 5–10 will be a promising biocontrol agent against banana wilt.

## Data Availability Statement

The sequences of strain 5–10 was deposited in the GenBank database with an accession number JACVYG000000000 and added this part of content in the manuscript.

## Author Contributions

TY and DZ developed the ideas and designed the experimental plans. JX and JH supervised the research. XZ, TJ, YC, DQ, KL, YZ, and WT performed some experiments. TY and MZ analyzed the data. TY and WW prepared the manuscript. All authors contributed to the article and approved the submitted version.

## Conflict of Interest

The authors declare that the research was conducted in the absence of any commercial or financial relationships that could be construed as a potential conflict of interest.

## Footnotes

1 http://trna.ucsc.edu/software/

2 http://trna.ucsc.edu/software/

3 https://github.com/tseemann/barrnap

4 https://www.ezbiocloud.net/identify

## Supplementary Material

Supplementary Figure 1 Effect of *Streptomyces* sp. 5–10 crude extracts on conidial germination of Foc RT4.

Supplementary Figure 2 Effect of extracts on mycelial morphology of Foc-GFP. (Scale bar = 200 μm).

Supplementary Table 1 Growth characteristics of *Streptomyces* sp. 5–10 on six media.

Supplementary Table 2 Physiological and biochemical characteristics of strain 5–10.

Supplementary Table 3 Predicted gene clusters in the strain 5–10 genome.

Supplementary Table 4 Plackett–Burman design for screening significant variables of fermentation condition of *Streptomyces* sp. 5–10.

Supplementary Table 5 Data analysis generated by the Plackett–Burman design.

Supplementary Table 6 Experimental design and results of the path of steepest ascent design.

Supplementary Table 7 Matrix and data of the BBD experiment.

Supplementary Table 8 ANOVA results of the regression equation.
