# Supplementary material for: Anti-Foc RT4 Activity of a Newly Isolated Streptomyces sp. 5–10 From a Medicinal Plant (Curculigo capitulata)
Source: Front Microbiol. 2021 Jan 22;11:610698. doi: 10.3389/fmicb.2020.610698 (PMC7862724; doi:10.3389/fmicb.2020.610698)
Supplement: Supplementary Figure 1 — Effect of Streptomyces sp. 5–10 crude extracts on conidial germination of Foc RT4. [file Data_Sheet_1.docx]

Supplementary Material


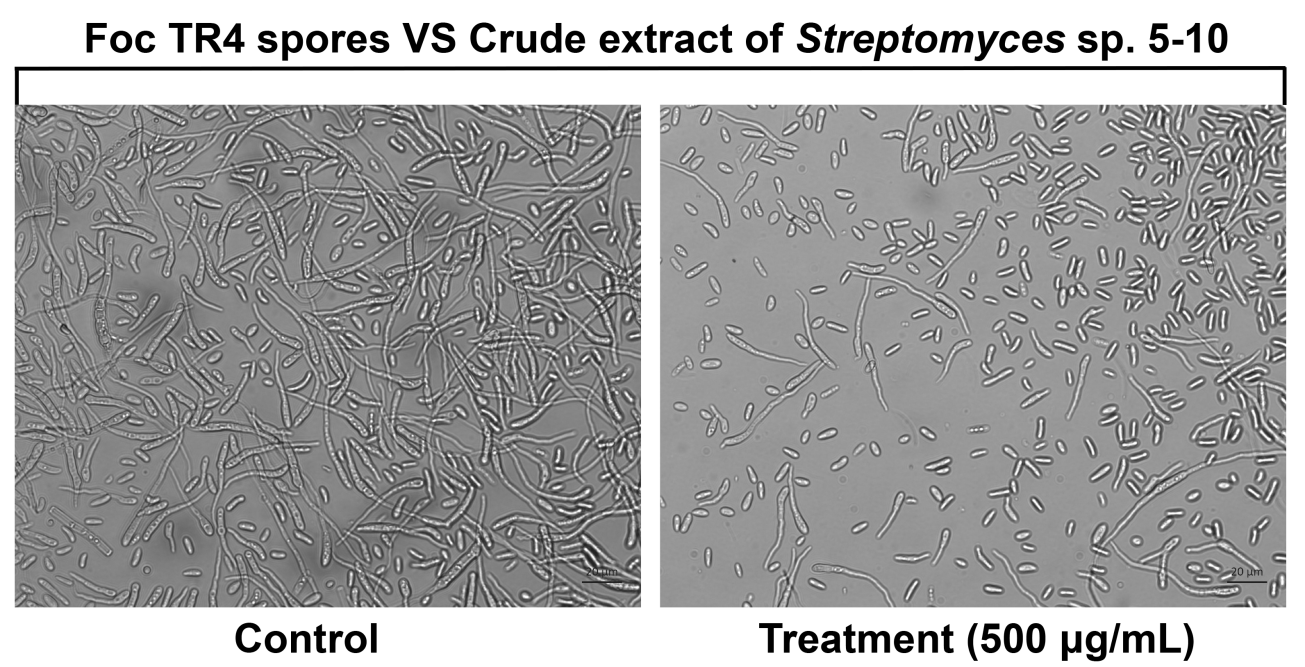


**Figure S1 |** Effect of *Streptomyces* sp. 5-10 extracts on conidial germination of Foc RT4.


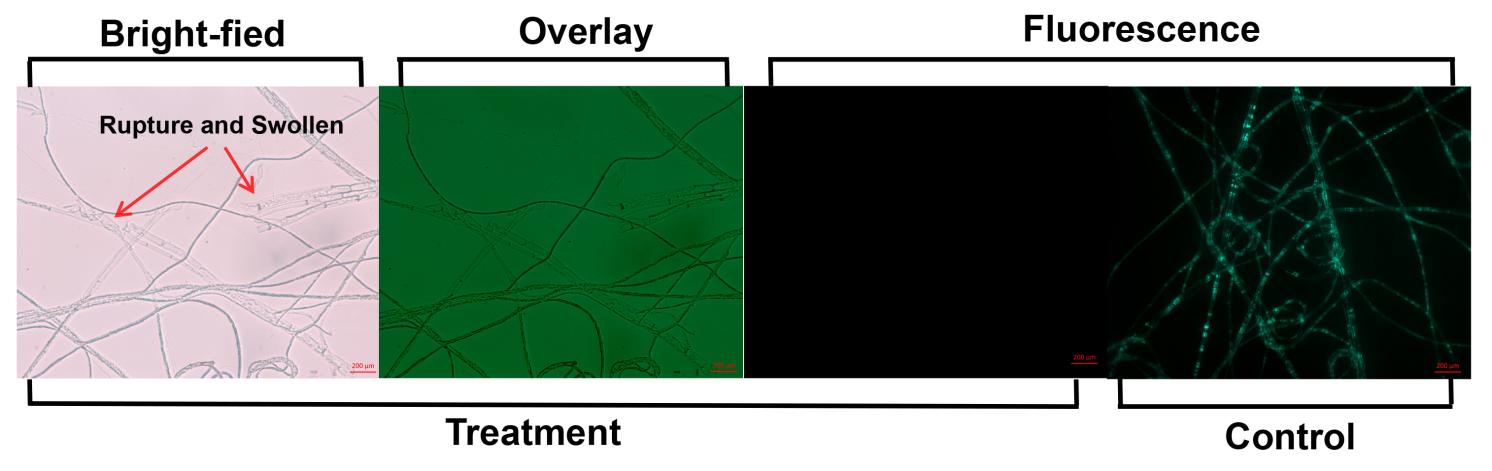


**Figure S2 |** Effect of extracts on mycelial morphology of Foc-GFP. Lyses effect of crude extract (red arrow), the Foc-GFP hypha became obvious coarse, rupture and fluorescence inactivation (Scale bar = 200 µm).

Supplementary Tables.

**Table S1 |** Growth characteristics of *Streptomyces* sp. 5-10 on six media

| Medium | Aerial mycelium | Kiene mycelium | Soluble pigment | Growth status | Colony morphology |
| --- | --- | --- | --- | --- | --- |
| Yeast malt extract agar (ISP2) | Wheat | Goldenrod | None | Good | 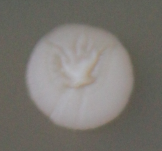 |
| Oat meal agar (ISP3) | White smoke | Khaki | None | Good | 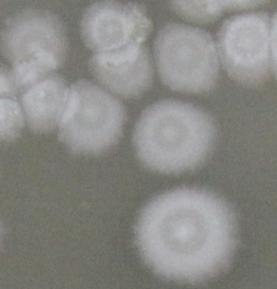 |
| Inorganic salt starch agar (ISP4) | Blanched almond | Khaki | None | Poor | 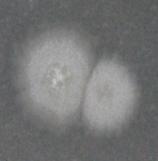 |
| Glycerol asparagine agar (ISP5) | White | Cornsilk | None | Poor | 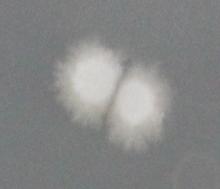 |
| Peptone yeast extract agar (ISP6) | Wheat | Goldenrod | None | Poor | 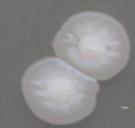 |
| Tyrosine agar (ISP7) | Blanched almond | Khaki | None | Moderate | 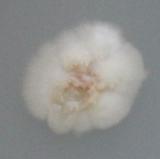 |

**Table S2 |** Physiological and biochemical characteristics of strain 5-10

| Characteristic | | Result |
| --- | --- | --- |
| BIOCHEMICAL TEST | | |
| Starch hydrolysis | | + |
| Gelatin hydrolysis | | + |
| Cellulose hydrolysis | | - |
| Nitratereduction | | + |
| H_2_S production | | - |
| Urease test | | + |
| Esterase test | Twain 20 | - |
|  | Twain 40 | - |
|  | Twain 80 | - |
| Protease test | | + |
| M-R test | | - |
| V-P test | | - |
| PHYSIOLOGICAL TEST | | |
| NaCl tolerance test (w/v) | | Up to 5% |
| pH range for growth | | 5-8 |
| NITROGEN SOURCE UTILIZATION | | |
| Glycine | | + |
| L-Phenylalanate | | + |
| Methionine | | + |
| Hydroxyproline | | + |
| Arginine | | - |
| Phenylalanine | | + |
| Histidine | | + |
| DL-methionine | | + |
| Cysteine | | + |
| Thiamine hydrochloride | | - |
| *α*-Naphthyl acid | | - |
| Tryptophan | | + |
| Serine | | + |
| Nicotinic acid | | - |
| Valine | | + |
| Tyrosine | | + |
| Asparagine | | + |
| Methionine | | + |
| Anhydrous creatine | | + |
| Glutamate | | - |
| CARBON-SOURCE UTILIZATION | | |
| L-Rhamnose | | - |
| D-Ribose | | + |
| Melezitose | | + |
| Melibiose | | + |
| Raffinose | | + |
| Xylan | | - |
| D-Xylose | | + |
| L-Arabinose | | - |
| Salicin | | + |
| D- Mannose | | + |
| D- Galactose | | + |
| D- Trehalose dihydrate | | + |
| Starch | | + |
| Sorbitol | | + |
| D-Fructose | | + |
| *α*- Lactose | | + |
| Glucose | | + |
| Cellobiose | | + |
| Sucrose | | + |
| D-Mannitol | | + |

Note: “+”, positive reaction; “−”, negative reaction.

**Table S3 |** Predicted gene clusters in the strain 5-10 genome

| Cluster ID | Cluster type | Start | End | Most similar known cluster | MIBiG accession | Compounds |
| --- | --- | --- | --- | --- | --- | --- |
| Cluster 1 | Terpene | 389,819 | 411,564 | Tiancilactone biosynthetic gene cluster from *Streptomyces* sp. CB03234 (17% of similarity) | BGC0002019 | Tiancilactone |
| Cluster 2 | NRPS | 3,712 | 63,087 | Coelichelin biosynthetic gene cluster from *Streptomyces coelicolor* A3(2) (90% of similarity) | BGC0000881 | Coelichelin |
| Cluster 3 | PKS-T1 | 395,217 | 433,473 | Azalomycin F3a biosynthetic gene cluster from *Streptomyces* sp. 211726 (78% of similarity) | BGC0001523 | Azalomycin F3a |
| Cluster 4 | PKS-T1 | 1 | 1,216 | - |  |  |
| Cluster 5 | PKS-T1 | 1 | 3,261 | - |  |  |
| Cluster 6 | NRPS-like | 1 | 1,320 | Rhizomide A biosynthetic gene cluster from *Paraburkholderia rhizoxinica* HKI 454 (100% of similarity) | BGC0000058 | Rhizomide A |
| Cluster 7 | NRPS | 1 | 4,719 | - |  |  |
| Cluster 8 | NRPS | 1 | 2,356 | - |  |  |
| Cluster 9 | NRPS | 1 | 12,659 | Cyclomarin D biosynthetic gene cluster from *Salinispora arenicola* CNS-205 (8% of similarity) | BGC0000333 | Cyclomarin D |
| Cluster 10 | NRP-Polyketide | 91,174 | 115,615 | Meridamycin biosynthetic gene cluster from *Streptomyces* sp. NRRL 30748 (44% of similarity) | BGC0001011 | Meridamycin |
| Cluster 11 | Terpene | 27,341 | 49,698 | Geosmin biosynthetic gene cluster from *Streptomyces coelicolor* A3(2) (100% of similarity) | BGC0001181 | Terpene |

**Table S5** (continued 1)

| Cluster ID | Cluster type | Start | End | Most similar known cluster | MIBiG accession | Compounds |
| --- | --- | --- | --- | --- | --- | --- |
| Cluster 12 | NRPS | 1 | 2,414 | Bicornutin A1 biosynthetic gene cluster from *Xenorhabdus budapestensis* (100% of similarity) | BGC0001135 | Bicornutin A1 Bicornutin A2 |
| Cluster 13 | NRPS-transat PKS-otherks | 1 | 79,340 | 2-methylisoborneol biosynthetic gene cluster from *Streptomyces griseus* subsp. *griseus* NBRC 13350 (100% of similarity) | BGC0000658 | 2-methylisoborneol |
| Cluster 14 | NRPS | 1 | 51,149 | Telomycin biosynthetic gene cluster from *Streptomyces canus* (5% of similarity) | BGC0001406 | Telomycin |
| Cluster 15 | PKS-T1 | 15,129 | 59,191 | Amipurimycin biosynthetic gene cluster from *Streptomyces novoguineensis* (86% of similarity) | BGC0001957 | Amipurimycin |
| Cluster 16 | NRPS | 58,039 | 102,157 | Ochronotic pigment biosynthetic gene cluster from *Streptomyces avermitilis* (50% of similarity) | BGC0000918 | Ochronotic pigment |
| Cluster 17 | Ectoine | 82,716 | 93,120 | Ectoine biosynthetic gene cluster from *Streptomyces anulatus* (100% of similarity) | BGC0000853 | Ectoine |
| Cluster 18 | PKS-T1 | 1 | 26,436 | Daptomycin biosynthetic gene cluster from *Streptomyces filamentosus* NRRL 11379 (10% of similarity) | BGC0000336 | Daptomycin |
| Cluster 19 | PKS-T1 | 1 | 4,421 | - |  |  |
| Cluster 20 | PKS-T2 | 1 | 69,199 | Spore pigment biosynthetic gene cluster from *Streptomyces avermitilis* (85% of similarity) | BGC0000271 | Spore pigment |
| Cluster 21 | Siderophore | 53,049 | 59,477 | - |  |  |

**Table S5** (continued 2)

| Cluster ID | Cluster type | | | Start | End | Most similar known cluster | | MIBiG accession | Compounds |
| --- | --- | --- | --- | --- | --- | --- | --- | --- | --- |
| Cluster 22 | | PKS-T1 | 1 | | 18,274 | | Naphthomycin A biosynthetic gene cluster from *Streptomyces* sp. CS (18% of similarity) | BGC0000106 | Naphthomycin A |
| Cluster 23 | | Terpene | 29,706 | | 50,806 | | Pristinol biosynthetic gene cluster from *Streptomyces pristinaespiralis* ATCC 25486 (100% of similarity) | BGC0001746 | Pristinol |
| Cluster 24 | | PKS-T1 | 10,356 | | 49,291 | | Elaiophylin biosynthetic gene cluster from Unknown. Unclassified (66% of similarity) | BGC0000053 | Elaiophylin |
| Cluster 25 | PKS-T1 | | | 1 | 9,970 | Nystatin biosynthetic gene cluster from *Streptomyces albulus* (31% of similarity) | | BGC0001709 | Nystatin |
| Cluster 26 | PKS-T1 | | | 1 | 30,989 | Elaiophylin biosynthetic gene cluster from Unknown. Unclassified (33% of similarity) | | BGC0000053 | Elaiophylin |
| Cluster 27 | PKS-T1 | | | 119,360 | 147,435 | Nigericin biosynthetic gene cluster from *Streptomyces violaceusniger* (83% of similarity) | | BGC0000114 | Nigericin |
| Cluster 28 | NRPS | | | 1 | 45,589 | Coelibactin biosynthetic gene cluster from *Streptomyces coelicolor* A3(2) (36% of similarity) | | BGC0000324 | Coelibactin |
| Cluster 29 | [Butyrolactone](https://docs.antismash.secondarymetabolites.org/glossary/#butyrolactone) | | | 91,134 | 101,991 | - | |  |  |
| Cluster 30 | Hserlactone | | | 1 | 8,535 | Heronamide A biosynthetic gene cluster from *Streptomyces* sp. SCSIO 03032 (8% of similarity) | | BGC0001349 | Heronamide A |
| Cluster 31 | NRPS-like | | | 1 | 28,1991 | Echoside A biosynthetic gene cluster from *Streptomyces* sp. LZ35 (94% of similarity) | | BGC0000340 | Echoside A |
| Cluster 32 | PKS-T1 | | | 1 | 26,941 | - | |  |  |

**Table S5** (continued 3)

| Cluster ID | Cluster type | Start | End | Most similar known cluster | MIBiG accession | Compounds |
| --- | --- | --- | --- | --- | --- | --- |
| Cluster 33 | PKS-T3 | 12,006 | 38,282 | Balhimycin biosynthetic gene cluster from *Amycolatopsis balhimycina* DSM 5908 (16% of similarity) | BGC0000311 | Balhimycin |
| Cluster 34 | Lassopeptide | 20,395 | 42,720 | SSV-2083 biosynthetic gene cluster from *Streptomyces sviceus* ATCC 29083 (36% of similarity) | BGC0000579 | SSV-2083 |
| Cluster 35 | PKS-T1 | 1 | 25,092 | Mediomycin A biosynthetic gene cluster from *Kitasatospora mediocidica* (50% of similarity) | BGC0001662 | Mediomycin A |
| Cluster 36 | PKS-T1 | 51,763 | 77,003 | ECO-02301 biosynthetic gene cluster from *Streptomyces aizunensis* (57% of similarity) | BGC0000052 | ECO-02301 |
| Cluster 37 | Arylpolyene | 10,794 | 50,543 | Atratumycin biosynthetic gene cluster from *Streptomyces atratus* (26% of similarity) | BGC0001975 | Atratumycin |
| Cluster 38 | PKS-T1 | 36,330 | 62,285 | s56-p1 biosynthetic gene cluster from *Streptomyces* sp. SoC090715LN-17 (11% of similarity) | BGC0001764 | s56-p1 |
| Cluster 39 | PKS-T1 | 1 | 1,309 | - |  |  |
| Cluster 40 | Arylpolyene | 1 | 64,954 | Atratumycin biosynthetic gene cluster from *Streptomyces atratus* (57% of similarity) | BGC0001975 | Atratumycin |
| Cluster 41 | Lassopeptide | 1,151 | 19,386 | - |  |  |
| Cluster 42 | PKS-T1 | 1 | 52,209 | Azalomycin F3a biosynthetic gene cluster from *Streptomyces* sp. 211726 (21% of similarity) | BGC0001523 | Azalomycin F3a |

**Table S5** (continued 4)

| Cluster ID | Cluster type | Start | End | Most similar known cluster | MIBiG accession | Compounds |
| --- | --- | --- | --- | --- | --- | --- |
| Cluster 43 | NRPS | 24,661 | 54,376 | - |  |  |
| Cluster 44 | NRPS | 1 | 3,035 | Rhizomide A biosynthetic gene cluster from *Paraburkholderia rhizoxinica* HKI 454 (100% of similarity) | BGC0001758 | Rhizomide A |
| Cluster 45 | Terpene | 1 | 18,575 | Hopene biosynthetic gene cluster from *Streptomyces coelicolor* A3(2) (61% of similarity) | BGC0000663 | Hopene |
| Cluster 46 | PKS-T1 | 1 | 57,654 | Tautomycin biosynthetic gene cluster from *Streptomyces spiroverticillatus* (20% of similarity) | BGC0000159 | Tautomycin |
| Cluster 47 | NRPS-like | 26,728 | 54,379 | Echoside A biosynthetic gene cluster from *Streptomyces* sp. LZ35 (94% of similarity) | BGC0000340 | Echoside A |
| Cluster 48 | PKS-T1 | 13,653 | 52,065 | Heronamide A biosynthetic gene cluster from *Streptomyces* sp. SCSIO 03032 (29% of similarity) | BGC0001349 | Heronamide A |
| Cluster 49 | NRPS-like | 1 | 24,939 | Streptolydigin biosynthetic gene cluster from *Streptomyces lydicus* (15% of similarity) | BGC0001046 | Streptolydigin |
| Cluster 50 | Indole | 14,903 | 36,060 | 5-isoprenylindole-3-carboxylate β-D-glycosyl ester biosynthetic gene cluster from *Streptomyces* sp. RM-5-8 (52% of similarity) | BGC0001483 | 5-isoprenylindole-3-carboxylate β-D-glycosyl ester |
| Cluster 51 | Siderophore | 1 | 11,020 | Desferrioxamin B biosynthetic gene cluster from *Streptomyces griseus subsp. griseus* NBRC 13350 (100% of similarity) | BGC0000941 | Desferrioxamin B |
| Cluster 52 | Iadderane | 4,157 | 39,452 | Atratumycin biosynthetic gene cluster from *Streptomyces atratus* (34% of similarity) | BGC0001975 | Atratumycin |
| Cluster 53 | NRPS | 9,037 | 34,511 | - |  |  |

**Table S5** (continued 5)

| Cluster ID | Cluster type | Start | End | Most similar known cluster | MIBiG accession | Compounds |
| --- | --- | --- | --- | --- | --- | --- |
| Cluster 54 | PKS-T1 | 1 | 22,430 | Sceliphrolactam biosynthetic gene cluster from *Streptomyces* sp. SD85 (52% of similarity) | BGC0001908 | Sceliphrolactam |
| Cluster 55 | PKS-T1 | 1 | 16,578 | Nigericin biosynthetic gene cluster from *Streptomyces violaceusniger* (83% of similarity) | BGC0000114 | Nigericin |
| Cluster 56 | PKS-T1 | 1 | 19,557 | Halstoctacosanolide A biosynthetic gene cluster from *Streptomyces halstedii* (77% of similarity) | BGC0000073 | Halstoctacosanolide A |
| Cluster 57 | PKS-T1 | 1 | 13,128 | Pladienolide B biosynthetic gene cluster from *Streptomyces platensis* (50% of similarity) | BGC0000126 | Pladienolide B |
| Cluster 58 | PKS-T1 | 1 | 14,898 | Angolamycin biosynthetic gene cluster from *Streptomyces eurythermus* (60% of similarity) | BGC0000018 | Angolamycin |
| Cluster 59 | NRPS | 1 | 2,302 | Luminmide biosynthetic gene cluster from *Photorhabdus laumondii subsp. laumondii* TTO1 (100% of similarity) | BGC0001128 | Luminmide |
| Cluster 60 | NRPS | 1 | 2,590 | Xenotetrapeptide biosynthetic gene cluster from *Xenorhabdus nematophila* ATCC 19061 (100% of similarity) | BGC0001132 | Xenotetrapeptide |

Note: “-” is no similar cluster predicted.

**Table S4 |** Plackett-Burman design for screening significant variables of fermentation condition *Streptomyces* sp. 5-10

| Run | Variables | | | | | | | | | Antifungal activity (%) |
| --- | --- | --- | --- | --- | --- | --- | --- | --- | --- | --- |
|  | X_1_ | X_2_ | X_3_ | X_4_ | X_5_ | X_6_ | X_7_ | X_8_ | X_9_ |  |
| 1 | 20 | 18.75 | 6.25 | 2.5 | 4 | 8 | 8 | 250 | 6 | 27.77 |
| 2 | 20 | 15 | 5 | 2 | 4 | 8 | 8 | 200 | 6 | 16.53 |
| 3 | 25 | 15 | 6.25 | 2.5 | 4 | 10 | 10 | 250 | 6 | 51.04 |
| 4 | 20 | 18.75 | 6.25 | 2 | 5 | 10 | 10 | 200 | 6 | 7.52 |
| 5 | 20 | 18.75 | 5 | 2.5 | 5 | 8 | 10 | 250 | 7.5 | 55.86 |
| 6 | 25 | 18.75 | 5 | 2 | 4 | 10 | 8 | 250 | 7.5 | 60.27 |
| 7 | 25 | 18.75 | 5 | 2.5 | 5 | 10 | 8 | 200 | 6 | 26.18 |
| 8 | 25 | 15 | 6.25 | 2.5 | 5 | 8 | 8 | 200 | 7.5 | 34.19 |
| 9 | 25 | 15 | 5 | 2 | 5 | 8 | 10 | 250 | 6 | 20.11 |
| 10 | 20 | 15 | 5 | 2.5 | 4 | 10 | 10 | 200 | 7.5 | 53.05 |
| 11 | 25 | 18.75 | 6.25 | 2 | 4 | 8 | 10 | 200 | 6 | 53.45 |
| 12 | 20 | 15 | 6.25 | 2 | 5 | 10 | 8 | 250 | 6 | 20.14 |

**Table S5 |** Data analysis generated by the Plackett-Burman design

| Coded  variable | Variable | DF | Parameter Estimate | F-value | P-value Prob > F | Significance |
| --- | --- | --- | --- | --- | --- | --- |
| Intercept | Constant | 1 | 35.50917 | 18.85 | 0.0028 | *** |
| X_1_ | Soluble starch | 1 | 5.36417 | 2.85 | 0.1044 |  |
| X_2_ | Soy flour | 1 | 2.99917 | 1.59 | 0.2524 |  |
| X_3_ | Yeast extract | 1 | -3.15750 | -1.68 | 0.2357 |  |
| X_4_ | Peptone | 1 | 5.83917 | 3.10 | 0.0902 |  |
| X_5_ | NaCl | 1 | -8.17583 | -4.34 | 0.0492 | * |
| X_6_ | pH | 1 | 0.85750 | 0.46 | 0.6936 |  |
| X_7_ | Time | 1 | 4.66250 | 2.47 | 0.1318 |  |
| X_8_ | Shaker speed | 1 | 3.68917 | 1.96 | 0.1893 |  |
| X_9_ | Inoculation amount | 1 | 10.65083 | 5.65 | 0.0299 | * |

Note: X_1_-X_9_ represent various variables. *p*-value of less than 0.05 indicates that the model terms are significant. The *t*-values indicate a positive or negative relationship of each factor. "+" or "-" represent a positive or negative effect on antifungal activity, respectively.

**Table S6 |** Experimental design and results of the path of steepest ascent design

|  | Variable | | Antifungal activity (%) |
| --- | --- | --- | --- |
|  | NaCl（g/L） | Inoculation amount（%） |  |
| 1. Test center point | 4.5 | 6.75 |  |
| 1. Test step size | 0.5 | 1.25 |  |
| (3) Slope | -16.35 | 21.30 |  |
| (4)Corresponding value size =(2)×(3) | -8.175 | 26.625 |  |
| (5) Step length =(4)×0.05^a^ | -0.409 | 1.331 |  |
| (6) Test No. 1 | 4.5 | 6.7 | 56.19 |
| No. 2 | 4.1 | 8.0 | 51.77 |
| No. 3 | 3.7 | 9.3 | 50.66 |
| No. 4 | 3.3 | 10.6 | 57.29 |
| No. 5 | 2.9 | 11.9 | 67.97 |
| No. 6 | 2.5 | 13.2 | 57.29 |

**Table S7 |** Matrix and data of the BBD experiment

| Run | Variable | | Antifungal activity (%) | | |
| --- | --- | --- | --- | --- | --- |
|  | NaCl (g/L) | Inoculation amount (%) | *Y1* (Test value) | *Y2* (Fit value) | Fitting error |
| 1 | -1 | 0 | 68.37 | 67.31 | 1.06 |
| 2 | 1 | 0 | 55.37 | 55.48 | -0.11 |
| 3 | 1 | -1 | 42.87 | 42.86 | 0.01 |
| 4 | 0 | 0 | 71.86 | 72.81 | -0.95 |
| 5 | 0 | 1 | 59.86 | 59.48 | 0.38 |
| 6 | 0 | -1 | 65.88 | 65.31 | 0.57 |
| 7 | -1 | 1 | 48.37 | 48.85 | -0.48 |
| 8 | -1 | -1 | 64.37 | 64.95 | -0.58 |
| 9 | 1 | 1 | 47.38 | 47.28 | 0.10 |

**Table S8 |** ANOVA results of the regression equation

| Source | DF | SS | MS | F-value | P-value  Prob>F |
| --- | --- | --- | --- | --- | --- |
| Model | 5 | 843.45 | 168.69 | 164.68 | 0.0007 |
| X_1_- NaCl | 1 | 209.92 | 209.92 | 204.94 | 0.0007 |
| X_2_-The inoculation amount | 1 | 51.10 | 51.10 | 49.89 | 0.0058 |
| X_1_ X_2_ | 1 | 105.17 | 105.17 | 102.67 | 0.0020 |
| X_1_^2^ | 1 | 260.45 | 260.45 | 254.26 | 0.0005 |
| X_2_^2^ | 1 | 216.81 | 216.81 | 211.65 | 0.0007 |
| Residual | 3 | 3.07 | 1.02 |  |  |
| Cor Total | 8 | 846.52 |  |  |  |
| Coefficient of determination  R^2^=0.9964 | | Adjusted coefficient of determination  R^2^=0.9903 | | Mean=58.26 | Coefficient of variation (CV)=1.74% |

Note: P> 0.05, insignificant difference; P <0.05, significant difference; P <0.01, significant difference.
